# Catching the threads: caregiving in Alzheimer's disease

**DOI:** 10.1192/bjp.2023.114

**Published:** 2023-11

**Authors:** Elizabeth Kuipers

**Affiliations:** Department of Psychology, School of Mental Health & Psychological Sciences, Institute of Psychiatry, Psychology & Neuroscience, King's College, London, UK.

**Keywords:** Dementias/neurodegenerative diseases, carers, psychosocial interventions, organic syndromes, stigma

## Abstract

As someone who has researched the effects on carers living with people with severe psychiatric disorders, the author describes her own recent experience of being a carer. The article serves as a companion piece to her psychiatrist husband's account of his cognitive decline in Alzheimer's disease.

My husband Paul Bebbington, an Emeritus Professor of Social and Community Psychiatry, wrote a brave and personal account^1^ about receiving a diagnosis of Alzheimer's at the beginning of 2022. The team at Croydon memory clinic were very good, as was the person who made a home visit from the Alzheimer's society; she was full of useful suggestions (e.g. not to leave things to the future, but to sort out whatever we needed to now). But it would have been containing to have a yearly follow-up from the memory clinic – currently not locally commissioned by the National Health Service (NHS). Offers of respite when needed would also have felt good. I am in the interesting position of having published research into caregiving for more than 40 years, and now needing to put it into practice for myself. As carers have always told us, the role is not for the fainthearted and not easy to get anywhere near right.

## Adapting to Paul's cognitive changes

The first signs of Paul having trouble, about 6 years ago, were relatively slight and not different from issues many friends and relatives have – misplacing keys, leaving travel cards at home. Irritating, but not very serious, and solved mainly by suggesting a ‘man bag’ with a shoulder strap, where important documents and wallet remained instead of the usual ransacking of every jacket pocket before going out. Later on, it became clear that some of the abstraction and conceptualising was more difficult. I could not suddenly change topic, and the usual shorthand for conversations between the married was not so easy. I had to be much more precise – not ‘Please get me the thingamy over there’, but ‘Please pick up the channel changer on the side table for me’. Being precise when in a hurry, or just busy with a grandchild or other tasks, is not my forte, but I am trying to improve.

Predicting what needs to be compensated for, without being overbearing, is another new requirement – a balance between stepping in and allowing for a few missteps. Predicting at all what will be problematic is in fact not predictable – ‘Get me the red cabbage’ (that we cut up and cooked yesterday and is down in our cellar fridge) resulted in ‘There is not a red cabbage down there’. I needed to ask for ‘the pot of cooked red cabbage that you put in the fridge for me last night’. Recently we went to New York and P went to buy a beer at the airport, until he realised he had left his ‘man bag’ – wallet and phone – in JFK security (which we did retrieve quite quickly). I meanwhile had been looking to check his shoes, as on a previous trip he had got to the other side of security in his socks, before asking me where his (only pair of) shoes were (in the X-ray queue).

## Becoming a carer

I had always, when asked, said to carers that they should never give up the day job – never make caregiving your only role in life. This is well evidenced as reducing perspective and being depressogenic.^2^ I had begun to slip into this – reducing my remaining work commitments, coming off review panels, saying ‘no’ to work events, as well as thinking about saying ‘no’ to looking after some of the grandchildren each week. It was only when I could not stop crying that it became clear that giving up my own interests was just narrowing my life. Making me feel defeated and a bit hopeless. I have put this right now – I need to accommodate both and find ways to manage while looking after P.

Although kindly meant, I could do without the *sotto voce* query from everyone ‘How is P?’ once he leaves the room. He is basically fine, not entirely fine, sad that things are more difficult for us both and not wishing to make things worse. Of course. But P also looks good, has fun socially, enjoys my cooking, lifts weights at the gym and still knows far better than most of us what all the capital cities are called. He has considerable cognitive reserve and knows and remembers many things, but there are now holes in the tapestry at unexpected moments.

## The research evidence and the reality

From the literature, particularly in psychosis, caregivers tell us that they need practical and emotional support and for the person they care for to get better.^3^ Many carers try ‘just hoping things will improve’. This is not so helpful: actively seeking help and problem-solving are better strategies. In dementia, emotion-focused coping and your appraisal of coping self-efficacy – that you will find a way to manage – are key predictors of better outcomes, because although delay is possible, the condition will not improve. I have the most wonderful extended blended family, and they frequently phone, Zoom, WhatsApp and turn up. The family principle is the line from *Crocodile Dundee* when there is a problem – tell one person, soon everybody knows, and there is no longer a problem. I also have lovely friends, neighbours and work colleagues, and it has been very helpful of P to be so open and publish about his diagnosis, so there are no secrets there either. Talking to our many grandchildren has been fine – we did not receive advice about this, but just saying that ‘Grandad is now having more trouble remembering things’ has worked well. One of them, who is 6, replied ‘Yes, I know, but I have a good memory’; he automatically rushed off and memorised our car registration when needed for the parking ticket. No problem!

But day-to-day caregiving is a lonely business, risking isolation and exhaustion and a few more glasses of chardonnay than recommended. P and I have put in place a 3-monthly review of how we are, use whiteboards and a paper diary (always open), and a routine for each day of the week, which I discuss each morning, now that P cannot always tell me what the date is. We have been members of a local choir for years and this is restorative. We started line dancing – you dance individually in lines, obviously – which we are rubbish at, but is great fun and an improvement on a previous attempt, some years back, at learning jive together – I had a great time whirling about with all the other chaps, while P was taken off for extra help in the corner …

The research is also clear about the importance of a good relationship. Having interviewed dementia caregivers,^4^ it was apparent that while good relationships could be maintained, poor ones tended to deteriorate, fuelled by blame and anger. Care was also more ‘burdensome’ if relationships were poor.^5^ So, we try to have fun and laugh about some of the absurdities. There are quite a few. Most of the problems are trivial – P loses something and I don't know where it is, so we buy another one, or look it up or whatever. My daughters used to go off with my hairbrushes (and my clothes), which often had a far better time out with them. In the end I had six hairbrushes. So far, so retrievable.

## The future

It is not clear how things will go. P is a large man and is becoming more clumsy – my pre-emptive touch and swerve are getting better. I cannot believe that he will not recognise me at some stage if I give him a clue. He is still driving, under advisement. He has a long reach and is very strong; gardening goes well. My colleague Professor Richard Brown shared our location on each of our phones – if the phones come too, we mainly know where we both are. Richard also suggested we do something memorable each week – so we do – art galleries, films, lunches with friends, weekends away. We travel – I look after all the documents – it is stressful but indeed memorable.

It is always a challenge to change roles, from clinical academic to someone with lived experience. It is not boring. I have always tried to act on evidence-based research – cold showers (short ones), not eating much, exercise. I am hoping to rise enough to this challenge. It is also tricky for equality in a relationship to start to move away. But stuff happens. Catching the threads while I can, and having a good time, with love, is only making me cry a bit.

## Data Availability

Data availability is not applicable to this article as no new data were created or analysed in this study.
